# Identifying Ovarian Cancer-Associated EV mRNA Expression Profiles Using Unsupervised Machine Learning and Non-Negative Matrix Factorization

**DOI:** 10.3390/bioengineering13060597

**Published:** 2026-05-22

**Authors:** Rama Krishna Thelagathoti, Chao Jiang, Dinesh S. Chandel, Wesley A. Tom, Cleo Sarmiento, Appolinaire Olou, Gary Krzyzanowski, M. Rohan Fernando

**Affiliations:** Molecular Diagnostic Research Laboratory, Center for Sensory Neuroscience, Boys Town National Research Hospital, Omaha, NE 68131, USA; ramakrishna.thelagathoti@boystown.org (R.K.T.); chao.jiang@boystown.org (C.J.); dinesh.chandel@boystown.org (D.S.C.); cleo.sarmiento@boystown.org (C.S.); appolinaire.olou@boystown.org (A.O.); gary.krzyzanowski@boystown.org (G.K.)

**Keywords:** extracellular vesicles, non-negative matrix factorization, ovarian cancer, EV mRNA transcriptomics, unsupervised machine learning, unsupervised feature selection

## Abstract

Extracellular vesicle (EV) transcriptomic data provides a high-dimensional representation of cellular states but remains challenging to interpret due to noise, redundancy, and limited sample sizes. Most existing approaches rely on supervised differential expression analyses, which can be biased and may fail to capture latent structure in small datasets. In this study, we propose an unsupervised machine learning framework based on non-negative matrix factorization (NMF) to identify latent expression programs from EV mRNA profiles. A structured preprocessing pipeline combining expression filtering, variance selection, ANOVA-based feature selection, and correlation pruning was used to reduce dimensionality and improve signal quality prior to matrix factorization. NMF was applied to decompose the data into interpretable gene modules and sample-specific activation patterns. Model selection was performed using reconstruction error and component stability across multiple initializations. Candidate features were prioritized using a composite ranking score integrating module loadings, group-level expression differences, and model stability. The approach identified a stable low-rank representation capturing dominant patterns in the data and a compact set of informative features. These results demonstrate that unsupervised matrix factorization can effectively extract structured, interpretable signals from small-scale transcriptomic datasets and provide a robust framework for feature prioritization and representation learning in high-dimensional biological data.

## 1. Introduction

Ovarian cancer is the most lethal gynecologic malignancy, with over 314,000 new cases and 207,000 deaths annually worldwide [1]. More than 70% of patients present at advanced stage, yielding five-year survival rates below 30% [2,3]. Extensive molecular heterogeneity encompassing at least four transcriptional subtypes in high-grade serous disease complicates treatment selection and has constrained progress in early detection [4,5]. In this context, tumor-derived EVs have emerged as informative molecular carriers. EVs are nanoscale, membrane-bound particles shed by tumor cells into peripheral biofluids, where their cargo mirrors the transcriptional and proteomic state of the originating tumor, offering a non-invasive window into tumor biology [6,7,8].

EVs carry a diverse RNA repertoire, including microRNAs, long non-coding RNAs, and messenger RNAs (mRNAs), the latter protected from extracellular ribonuclease degradation by the vesicular lipid bilayer [9,10]. EV mRNA packaging is not passive; selective sorting mechanisms enrich specific transcripts relative to the donor cell, suggesting that EV mRNA profiles faithfully encode biologically meaningful transcriptional states [11,12]. Prior EV studies in ovarian cancer have focused predominantly on individual transcripts or small panels evaluated through supervised differential expressions, yielding important but necessarily narrow insights [13,14,15]. Transcriptional regulation in cancer is organized into co-expressed gene modules reflecting shared pathway activation and regulatory architecture [16,17], yet whether such coordinated programs are recapitulated within the EV mRNA compartment remains poorly characterized. The systems-level organization of EV mRNA expression in ovarian cancer thus represents a significant and underexplored dimension of EV biology.

Standard RNA-seq pipelines model per-gene count distributions under predefined group contrasts as implemented in DESeq2 and edgeR testing features independently and ignoring the multivariate covariance structure of the expression matrix [18,19]. In high feature-to-sample regimes typical of EV datasets, this univariate, label-dependent design is prone to inflated error rates and cannot recover signals distributed across co-varying gene sets [20]. NMF addresses this directly by learning a low-rank, parts-based decomposition of the full expression matrix without reference to sample labels: non-negativity constraints produce additive, sparse components that map naturally to co-expressed gene programs, and rank selection is guided by reconstruction error and cophenetic correlation across random initializations [21,22,23]. Applied across bulk and single-cell cancer datasets, NMF has reliably recovered mutational signatures, cell-state programs, and molecular subtypes [24,25], yet its use on EV mRNA matrices remains unreported.

We hypothesized that the EV mRNA expression matrix in ovarian cancer harbors latent low-rank structure encoding tumor-associated transcriptional programs recoverable by NMF without supervision. The pipeline comprised four stages: (i) expression- and variance-based row filtering followed by ANOVA F-statistic feature selection and pairwise correlation pruning to reduce input dimensionality; (ii) NMF rank sweep with cophenetic stability-based rank selection; (iii) identification of cancer-associated components by Cohen’s d effect size on per-sample activation scores, with significance assessed by permutation testing under label-shuffled nulls; and (iv) candidate gene scoring integrating W-matrix loading weight, between-group expression delta, and bootstrap resampling stability. The result is a fully unsupervised, label-free gene ranking orthogonal to supervised differential expression, intended as a computational discovery framework rather than a validated pipeline.

## 2. Related Work

The application of unsupervised matrix factorization to cancer transcriptomics has gained considerable traction as a principled alternative to label-dependent statistical tests. Gavish et al. applied NMF across single-cell RNA-seq data from over 1000 tumor samples spanning 24 cancer types, demonstrating that per-sample factorizations converge on a reproducible set of consensus meta-programs when aggregated via similarity clustering, establishing both the biological generalizability and the computational stability of NMF-derived gene modules in oncology [25]. A systematic review by Hamamoto et al. further benchmarked NMF against PCA across a range of omics tasks, concluding that the non-negativity constraint makes NMF uniquely suited to transcriptomic data where signals are additive and genes participate in overlapping regulatory programs; the review explicitly catalogues ovarian cancer among the cancer types where NMF-based decomposition has demonstrated analytical value [23]. Together, these studies provide the computational rationale for applying NMF to the EV mRNA expression matrix in the present work, and inform the rank selection and resampling stability framework used to validate recovered components.

Within the EV field, however, unsupervised data-driven approaches remain underutilized. A comprehensive methods review by Miceli et al. surveyed the full bioinformatic stack for EV RNA-seq analysis and identified the absence of structure-aware, label-free analytical frameworks as a key methodological gap, particularly for the low-sample, high-feature regime that characterizes most EV transcriptomic datasets [26]. In the clinical domain, Jo et al. developed an EV-based liquid biopsy panel for ovarian cancer detection using a supervised, hypothesis-driven proteomic strategy, achieving promising classification performance but acknowledging that the approach captures only a narrow slice of EV cargo complexity and does not address the global transcriptional organization of tumor-derived EVs [27]. The closest computational precedent for the present work is Dogra et al., who applied an annotation-agnostic unsupervised pipeline to EV small RNA-seq data from prostate cancer patients and recovered biologically meaningful latent signals termed transcriptional dark matter that were not detectable by supervised differential expression [28]. Collectively, these studies delineate the methodological gap that the present work addresses: the lack of an unsupervised, latent-structure framework for EV mRNA transcriptomics in ovarian cancer.

## 3. Materials and Methods

### 3.1. Experimental Design

#### 3.1.1. Cell Culture, EV Isolation, and RNA Extraction

Human ovarian cancer cell lines SKOV3, TOV21G, and OVCAR-3 were purchased from the American Type Culture Collection (ATCC, Manassas, VA, USA). Ovarian cancer cell lines IGROV-1 and COV362.4 were obtained from Sigma-Aldrich (St. Louis, MO, USA). The OVCAR-5 cell line was purchased from Cell Biolabs (San Diego, CA, USA), and OVCAR-8 cells were obtained from Creative Biolabs (Shirley, NY, USA). The immortalized human normal ovarian epithelial cell line HOSE-T80 was generously provided by Dr. John Davis (University of Nebraska Medical Center, Omaha, NE, USA). Immortalized human normal ovarian epithelial cells HIO-80 were kindly provided by Dr. Andrew K. Godwin (University of Kansas Medical Center, Kansas City, KS, USA). Human normal ovarian epithelial cells (HOSE-T80 and HIO-80) were cultured in a 1:1 mixture of Medium 199 and MCDB105. All ovarian cancer cell lines (SKOV3, TOV21G, OVCAR-3, IGROV-1, COV362.4, OVCAR-5, and OVCAR-8) were maintained in Dulbecco’s Modified Eagle Medium (DMEM). All culture media were supplemented with 10% fetal bovine serum (FBS) and penicillin–streptomycin (100 μg/mL). Cells were incubated at 37 °C in a humidified atmosphere containing 5% CO_2_.

When the cells reached approximately 60% confluency, the culture medium containing 10% FBS was replaced with medium supplemented with 10% exosome-depleted FBS (Thermo Fisher Scientific; cat. no. A2720803, Waltham, MA, USA) to minimize contamination from serum-derived EVs [29]. Conditioned media were collected 48–72 h later for EV isolation. The collected culture medium was first centrifuged at 2000× *g* for 30 min to remove cells and cellular debris. The resulting supernatant was used for EV isolation using differential ultracentrifugation, a widely used method for EV purification [7,30]. Briefly, the cell- and debris-free media were centrifuged at 100,000× *g* for 3 h. The EV pellet was washed with 1× phosphate-buffered saline (PBS) followed by a second ultracentrifugation under the same conditions. The final EV pellet was resuspended in PBS and either used immediately or stored at −80 °C for downstream analysis.

Total RNA was extracted from the EV pellet using the GeneJET™ RNA Purification Kit (cat. no. K0731; Thermo Fisher Scientific, Waltham, MA, USA) according to the manufacturer’s instructions. Briefly, EV pellets were lysed and RNA was purified using silica-membrane columns to obtain total RNA suitable for downstream transcriptomic analysis.

#### 3.1.2. EV RNA-Seq Processing and Quantification

Total RNA was extracted from EVs derived from all cell lines in triplicate and assessed for quality and purity using a Qubit High Sensitivity RNA assay (Thermo Fisher Scientific, Waltham, MA, USA) and a High-Sensitivity RNA TapeStation assay (Agilent Technologies, Santa Clara, CA, USA). All samples exhibited RNA integrity number (RIN) values ≥ 5.5. Library preparation was performed using Novogene’s ultra-low input total RNA directional library preparation protocol with ≥25 ng of EV RNA (Novogene Corporation, Sacramento, CA, USA). Ribosomal RNA (rRNA) was depleted from total RNA to enrich for non-ribosomal transcripts, specifically 28S, 18S, 5.8S, and 5S cytoplasmic rRNAs 16S and 12S mitochondrial rRNAs, 45S ETS and ITS rRNAs, HBA1/2, HBB, HBD, HBM, HBG1/2, HBE1, HBQ1, and HBZ globin RNAs. This depletion enriched for long non-coding RNAs (lncRNAs) and messenger RNAs (mRNAs). The remaining RNA was fragmented and reverse-transcribed to generate first-strand cDNA, followed by second-strand synthesis to produce strand-specific libraries. After end repair, A-tailing, and adaptor ligation, libraries were PCR-amplified and purified. Indexed libraries were sequenced on an Illumina NovaSeq 6000 platform using paired-end 150 bp reads (PE150) (Illumina Inc., San Diego, CA, USA), generating approximately 12 Gb of raw data per sample.

Extracellular vesicle RNA-seq data were processed using a custom bioinformatic pipeline. Raw FASTQ files underwent quality control using FastQC [31], followed by adapter and quality trimming with BBDuk from the BBMap suite [32]. Trimmed reads were aligned to the human reference genome GRCh38 using STAR [33] with GENCODE v44 annotations [34], employing two-pass mapping and parameters optimized for short EV-derived RNA fragments. Post-alignment quality metrics, including alignment statistics, insert size distributions, and duplicate rates, were computed using SAMtools [35] and Picard Tools [36]. Transcript-level abundances across all RNA biotypes were estimated using Salmon, employing bias-aware quasi-mapping with GC and sequence bias correction [37]. Per-sample quantifications were merged using Salmon’s quantmerge functionality to generate a unified transcripts-per-million (TPM) expression matrix for downstream analyses.

**Figure 1:**
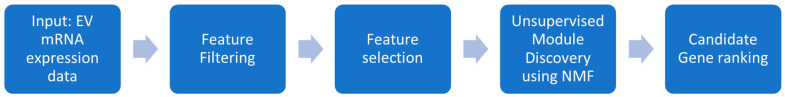
Analytical workflow for identification of ovarian cancer-associated EV mRNA signatures. Initial feature filtering removed low-expression and low-variance transcripts, followed by supervised feature selection to prioritize cancer-informative genes. Non-negative matrix factorization (NMF) was then applied to identify latent expression modules. Genes within the cancer-associated module were subsequently ranked based on module contribution and differential expression to derive a prioritized gene panel.

The overall analytical workflow used for identifying ovarian cancer-associated EV mRNAs is illustrated in Figure 1.

### 3.2. Feature Filtering and Dimensionality Reduction

To reduce noise and improve the stability of downstream matrix factorization, two sequential feature filtering steps were applied to the genes-by-samples expression matrix prior to modeling.

- *Expression filtering*: Genes with negligible or sporadic expression across the sample set were removed to eliminate low-information and unreliably detected transcripts. A gene was retained only if its normalized expression exceeded a minimum count threshold of 7 in at least 2 samples. This criterion excludes transcripts whose apparent signal likely reflects stochastic sequencing noise, sample-specific dropout, or library preparation artefacts, while preserving genes with reproducible, detectable expression across the dataset [38]. Retaining only consistently expressed features improves the signal-to-noise ratio of the input matrix and enhances numerical stability for subsequent factorization.
- *Variance-based feature selection*: Following expression filtering, genes were ranked by their across-sample variance and the top 7000 genes were retained. Features with high cross-sample variance are more likely to encode biologically meaningful differences between ovarian cancer and control samples [39]. Together, these two steps produced a compact, high-signal feature matrix enriched for consistently expressed and biologically variable transcripts, constituting the input to the ANOVA-based feature selection and unsupervised matrix factorization stages described below.

### 3.3. Feature Selection

To further refine the feature space and prioritize genes associated with ovarian cancer state, a combination of supervised and unsupervised statistical feature selection strategy was applied following initial preprocessing.

ANOVA F-Test-Based Selection: An analysis of variance (ANOVA) F-test was performed to identify genes exhibiting differential expression between ovarian cancer and control samples. For each gene, the F-statistic was computed to quantify the ratio of between-group variance to within-group variance, thereby assessing the degree to which gene expression differed across phenotypic classes [40]. Genes were ranked according to their F-statistic values, and the top-ranked genes were retained for downstream analysis. This approach enabled prioritization of features that contribute most strongly to ovarian–control separation while preserving a sufficiently large feature set to maintain systems-level structure.

Correlation-Based Redundancy Filtering: To reduce redundancy and minimize the influence of highly correlated features, unsupervised correlation-based pruning was subsequently applied. The Pairwise Pearson correlation coefficients were calculated across all retained genes using their expression profiles across samples. Genes exhibiting strong pairwise correlation (absolute correlation coefficient |r| > 0.95) were considered redundant, and one gene from each highly correlated pair was removed using a greedy selection strategy [41]. This step reduces multicollinearity, improves model stability, and ensures that the final feature set captures diverse biological signals rather than duplicated expression patterns.

Together, ANOVA-based prioritization and correlation-based pruning yielded a compact, biologically informative feature matrix optimized for robust NMF and downstream module discovery.

### 3.4. Non-Negative Matrix Factorization (NMF)

To identify latent gene expression programs within the EV mRNA dataset, we applied NMF, an unsupervised dimensionality reduction technique designed for non-negative data matrices [42]. NMF decomposes a gene expression matrix into a set of latent components representing groups of co-expressed genes.

Let $X\in R_{\geq 0}^{g\times n}$ denotes the EV mRNA expression matrix where

*g* represents the number of genes;

*n* represents the number of samples.

NMF approximates this matrix as the product of two lower-dimensional non-negative matrices:

X≈WH

where

$W\in R_{\geq 0}^{g\times k}$ is the gene–module matrix;

$H\in R_{\geq 0}^{k\times n}$ is the module–sample activation matrix;

k is the number of latent components (modules).

**Figure 2:**
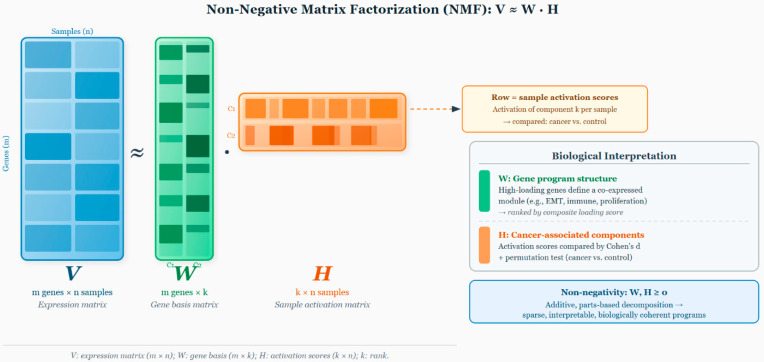
Conceptual framework of non-negative matrix factorization (NMF) applied to EV mRNA expression data. The gene-by-sample expression matrix V (*m* genes × *n* samples) is factorized into two non-negative matrices: the gene basis matrix W (*m* × *k*), representing latent gene programs, and the sample activation matrix H (*k* × *n*), representing module activity across samples. Columns of W define co-expressed gene modules, while rows of H quantify component activation in each sample, enabling comparison between ovarian cancer and control groups.

As illustrated in Figure 2, this factorization separates the original gene expression matrix into two biologically interpretable components:

Gene modules (W): Each column of W represents a set of genes that tend to be co-expressed across samples. The values in this matrix correspond to gene loadings, which quantify how strongly each gene contributes to a specific module. Genes with high loading values define the core members of a biological program.

Module activation scores (H): Each row of H describes the activation level of a module across all samples. These values indicate how strongly each gene program is expressed in individual samples.

Using this representation, gene expression patterns can be interpreted as combinations of a small number of underlying transcriptional programs. In the present study, genes within each module were ranked according to their loading weights in W and a downstream candidate gene score. Module activation values from H were then compared between ovarian cancer and control samples. To quantify the association of each module with cancer state, we calculated Cohen’s d-like effect size between groups and evaluated statistical significance using permutation testing [43].

The optimal number of latent components (k) for NMF was determined by jointly evaluating reconstruction error and component stability. For each candidate value of k, NMF was performed using multiple random initializations. Model fit was quantified using the Frobenius reconstruction error:

$$∥V-WH{∥}_{F}^{2}$$

where V is the original gene expression matrix and WH represents its low-rank approximation. As expected, reconstruction error decreased with increasing k; however, larger values of k increase model complexity and risk overfitting.

To evaluate robustness, component stability was assessed by aligning the gene-loading matrices (W) obtained from repeated NMF runs and calculating the mean absolute correlation between matched components. Stable components indicate reproducible gene modules across random initializations. The optimal k was selected as the smallest value that achieved high stability while maintaining low reconstruction error and biological interpretability. This strategy ensured robust identification of latent EV mRNA programs while maintaining model parsimony given the limited sample size [29].

Unlike principal component analysis (PCA) [44], NMF imposes non-negativity constraints:

W,H≥0

which produce additive, parts-based representations that are more biologically interpretable [45,46]. This property is particularly appropriate for transcriptomic data, where gene expression values are inherently non-negative and biological processes typically reflect coordinated activation of genes rather than opposing directions. The non-negative constraint therefore promotes sparse and interpretable gene modules, facilitating the identification of coherent transcriptional programs associated with ovarian cancer.

### 3.5. Gene Ranking and Candidate Prioritization

To identify the most informative genes within the cancer-associated NMF module, we developed a candidate ranking strategy that integrates three key aspects: (i) the structural importance of a gene within the NMF module, (ii) its association with the ovarian cancer phenotype, and (iii) the stability of the model across repeated runs. Gene ranking was restricted to the module that showed the strongest association with ovarian cancer, as determined by module effect size and permutation testing.

Candidate Gene Ranking Score:

For each gene i in the selected module k, a candidate ranking score was calculated as:

$${\mathrm{Score}}_{i}=|W_{i,k}|\times |\Delta_{k}|\times |{log}_{2}FC_{i}|\times S$$

where:

$W_{i,k}$ is the loading of gene i in module k from the NMF gene–module matrix W. Higher values indicate stronger contribution of the gene to that module.

$\Delta_{k}$ represents the effect size of module k between ovarian cancer and control samples.

${log}_{2}FC_{i}$ is the gene-level ${log}_{2}$ fold change between ovarian and control samples.

S is a stability factor that measures how consistently the gene appears across repeated NMF runs with different random initializations.

Module Effect Size:

The module effect size $\Delta_{k}$ quantifies how strongly the module separates ovarian and control samples and was calculated as:

$$\Delta_{k}=\frac{\mu_{\mathrm{ovarian}}-\mu_{\mathrm{control}}}{\sigma_{k}}$$

where:

$\mu_{\mathrm{ovarian}}$ and $\mu_{\mathrm{control}\;}$ represent the mean module activity values derived from the matrix H;

$\sigma_{k}$ is the pooled standard deviation of the module activity.

Gene-Level Expression Difference:

The gene-level log fold change was computed as:

$${log}_{2}FC_{i}={log}_{2}\left(\frac{{\bar{X}}_{i,\mathrm{ovarian}}+1}{{\bar{X}}_{i,\mathrm{control}}+1}\right)$$

where:

$\bar{X}$ denotes the mean expression of gene i within each group;

A pseudo count of 1 was added to avoid division by zero.

Candidate Gene Prioritization:

Genes were ranked in descending order of the candidate score, and the highest-scoring genes were considered candidate ovarian cancer-associated EV transcripts for downstream biological interpretation.

This multiplicative scoring framework prioritizes genes that simultaneously satisfy four criteria:

- Strong contribution to the cancer-associated module;
- Presence in a module that clearly distinguishes ovarian cancer from controls;
- Substantial differential expression between groups;
- Consistency across repeated NMF runs.

By integrating unsupervised module structure, phenotypic association, and model robustness, this strategy prioritizes biologically meaningful and reproducible candidate transcripts rather than genes identified solely by differential expression or module loading alone.

## 4. Results

The raw EV mRNA expression matrix contained approximately 186,000 transcripts across nine samples. To reduce technical noise and improve model stability, a structured multi-stage filtering strategy was applied. First, expression filtering retained genes with measurable expression in at least two samples, reducing the dataset to 10,003 genes. Next, variance-based selection was applied to retain the 6000 most variable genes, enriching the dataset for biologically informative transcripts. To further prioritize genes associated with ovarian cancer state, an ANOVA F-test was performed and the top 5000 genes ranked by F-statistic were retained. Finally, correlation-based redundancy pruning (|r| > 0.995) was used to remove highly correlated transcripts, resulting in a compact feature matrix of 3000 genes for downstream analysis.

NMF was applied to this filtered matrix to identify latent EV mRNA expression programs. Model selection was performed by evaluating reconstruction error and component stability across candidate values of K=2 to 6. As shown in Table 1, reconstruction error decreased progressively with increasing K, while stability metrics remained maximal across runs. The optimal number of components was selected by ranking models according to highest stability and lowest reconstruction error across repeated random initializations. Based on these criteria, K=6 was selected for downstream analysis. In addition, inspection of module composition indicated that K = 6 yielded distinct, non-overlapping gene programs, whereas lower K values resulted in merged or less specific modules.

To determine which latent component was associated with ovarian cancer state, module activity differences between ovarian and control samples were quantified using a Cohen-like effect size (Table 2). Among the six modules, Module 5 exhibited the strongest separation between groups with an effect size of −1.366, indicating a pronounced difference in activity between ovarian and control samples. Because this module demonstrated the largest magnitude of ovarian–control divergence, Module 5 was designated as the cancer-associated module for downstream gene prioritization.

Genes within the cancer-associated module were then ranked using a candidate scoring framework integrating module loading, gene-level log_2_ fold change, and module effect size. After applying ranking thresholds and filtering criteria, 87 candidate genes were retained. The highest-ranking genes included several small nuclear RNAs and ribosomal transcripts, such as RNVU1-7, RNU1-1, RN7SL1, RN7SK, and RN7SL3, as well as translational and structural genes including RPL41, RPL12, RPS20, RPS15A, RPS2, and RPL19. Additional biologically relevant genes included FTH1, which exhibited a large log_2_ fold change, and ANP32B, a regulator of chromatin structure and cell proliferation. Several genes demonstrated particularly strong ovarian–control differences, including RNVU1-7 (log_2_FC = 8.97), RNU1-1 (log_2_FC = 3.43), and FTH1 (log_2_FC = 3.91), indicating robust phenotypic separation between groups. This candidate ranking approach ensured that prioritized genes were both structurally central to the cancer-associated module and differentially expressed between ovarian and control samples.

**Figure 3:**
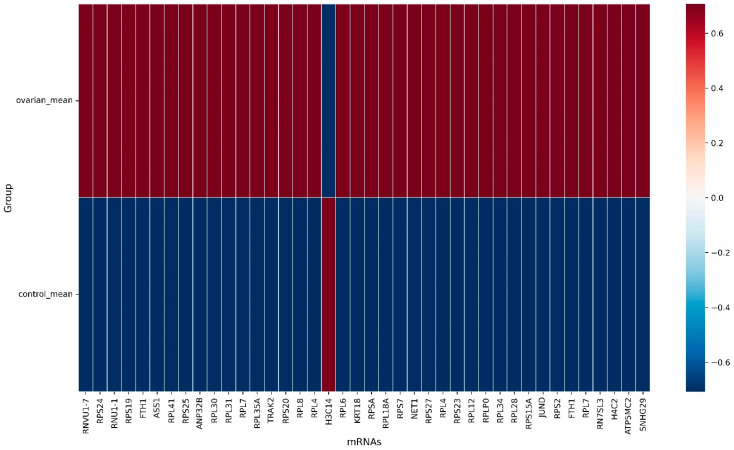
Heatmap of the top 40 EV mRNA panel differentiating ovarian cancer from controls. Row-wise z-score–standardized expression values (log-transformed) of the top 40 ranked EV mRNAs are shown across ovarian cancer and control samples. Genes are displayed as columns and sample groups as rows. Red indicates relatively higher expression, and blue indicates lower expression within each gene. The heatmap demonstrates coordinated overexpression of the majority of transcripts in ovarian samples compared with controls, highlighting a dominant cancer-associated EV transcriptional program.

**Figure 4:**
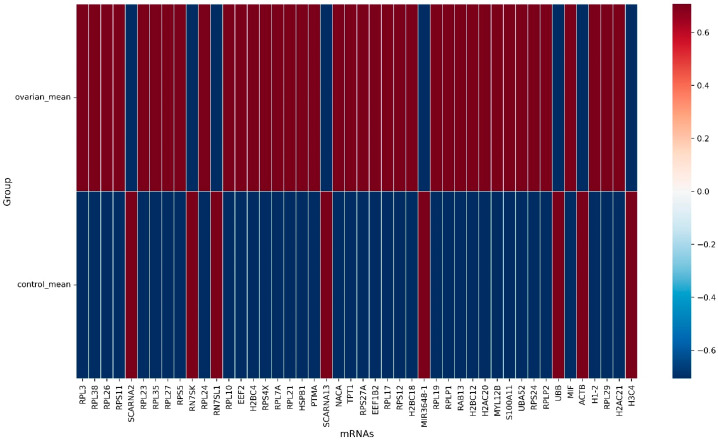
Heatmap of the remaining EV mRNA panel differentiating ovarian cancer from controls. Row-wise z-score–standardized (log-transformed) expression profiles of the remaining EV mRNA candidates are shown across ovarian cancer and control samples. Genes are displayed as columns and sample groups as rows. Red indicates relatively higher expression and blue indicates lower expression within each gene. Consistent with the primary panel, most transcripts exhibit elevated expression in ovarian samples, while a small subset shows relative downregulation, reinforcing the directional bias of the cancer-associated EV expression program.

Expression patterns of the prioritized transcripts are illustrated in Figure 3 and Figure 4, which display row-wise z-score–standardized heatmaps of the candidate EV mRNAs. The majority of genes showed coordinated overexpression in ovarian samples relative to controls, revealing a consistent cancer-associated EV transcriptional signature.

Notably, the EV mRNA panel exhibited a strong directional bias toward increased expression in ovarian samples. Among the 87 candidate transcripts, only one gene showed clear under expression within the top-ranked subset (Figure 3), and only eight genes displayed reduced expression among the next 47 candidates (Figure 4). This pronounced asymmetry suggests that the identified module reflects an activated transcriptional program rather than gene suppression. The coordinated upregulation of ribosomal proteins, translational regulators, and metabolic genes further supports the interpretation that ovarian cancer cells selectively package transcripts associated with proliferation and metabolic adaptation into EVs. Collectively, these findings highlight a coherent EV mRNA expression program associated with ovarian cancer. In addition to the averaged ovarian-versus-control heatmaps presented in the main figures, the expression patterns of all 87 candidate mRNAs across individual ovarian cancer and control cell lines are provided in Supplementary Figure S1, allowing visualization of inter-sample variability and transcript consistency across individual cell lines.

Among the identified EV mRNA candidates, several genes have previously established links to cancer biology, ovarian cancer progression, extracellular vesicle signaling, or translational regulation, supporting the biological relevance of the detected module. Notably, ASS1 has been strongly associated with arginine metabolism and arginine-deprivation vulnerability in ovarian and other cancers, while FTH1 is linked to iron homeostasis, ferroptosis regulation, platinum resistance, and ovarian cancer malignancy [47,48]. Additional biologically relevant candidates include KRT18, an epithelial cytoskeletal marker frequently associated with epithelial ovarian tumors; HSPB1, a stress-response chaperone implicated in tumor survival and chemoresistance; MIF, a pro-inflammatory cytokine involved in ovarian cancer progression; and JUND, a component of AP-1 transcriptional signaling linked to cellular proliferation and oncogenic stress responses. The enrichment of multiple ribosomal and translational genes, including RPS24, RPS19, RPL41, RPL30, and EEF2, is also consistent with prior reports demonstrating that increased ribosome biogenesis and translational activation are hallmarks of cancer progression [49,50]. Furthermore, EV-associated small RNA transcripts such as RNU1-1, RN7SL1, RN7SL3, and RN7SK are consistent with previous evidence that extracellular vesicles selectively transport diverse RNA species involved in intercellular communication [7,9]. In contrast, several highly ranked transcripts, particularly RNVU1-7, SNHG29, SCARNA13, and MIR3648-1, remain relatively underexplored in ovarian cancer EV biology and may represent potentially novel EV-associated candidates requiring further validation.

### 4.1. Pathway Enrichment Analysis of Cancer-Associated EV mRNAs

**Figure 5:**
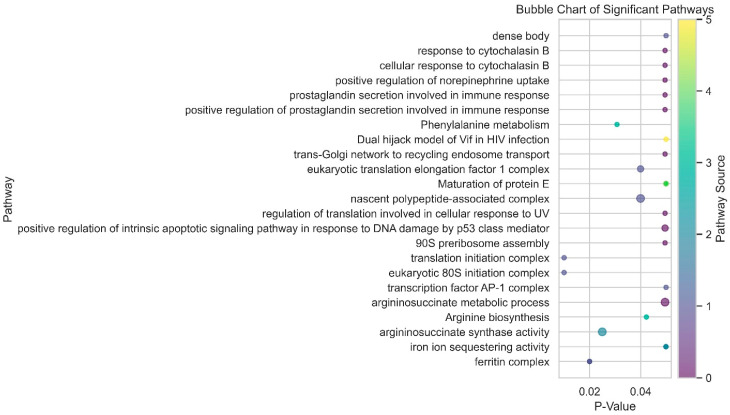
Bubble plot of significantly enriched biological pathways associated with the ovarian cancer-related EV mRNA program. Significant pathways identified through Gene Ontology (GO), KEGG, Reactome, and WikiPathways enrichment analyses are displayed. The x-axis represents nominal *p*-values, with smaller values indicating stronger enrichment. Bubble size corresponds to the relative enrichment strength (e.g., gene overlap or enrichment magnitude), and color denotes the source database. Enriched pathways highlight processes related to iron homeostasis (ferritin complex, iron ion sequestration), arginine metabolism, ribosome assembly and translation initiation, apoptotic signaling, and inflammatory regulation, supporting the biological relevance of the identified EV expression module.

To elucidate the biological programs represented by the ovarian cancer-associated EV mRNA panel, functional enrichment analysis was performed using Gene Ontology (GO), KEGG, Reactome, and WikiPathways databases (shown in Figure 5). Significant enrichment (*p* < 0.05) revealed coordinated involvement of iron metabolism, amino acid biosynthesis, translational regulation, apoptotic signaling, and inflammatory processes. Among the most significant terms were enrichment of the ferritin complex (GO:0070288) and iron ion sequestering activity (GO:0140315). Ferritin-mediated iron storage is tightly linked to tumor proliferation, oxidative stress tolerance, and ferroptosis resistance in ovarian cancer [51,52]. Elevated ferritin expression has been reported in epithelial ovarian tumors and is associated with aggressive disease phenotypes [51]. The enrichment of iron sequestration pathways suggests that EV-derived transcripts may reflect metabolic reprogramming that supports tumor growth and redox homeostasis.

Metabolic pathways were further represented by enrichment of argininosuccinate synthase activity (GO:0004055) and arginine biosynthesis (KEGG:00220). Arginine metabolism is increasingly recognized as a vulnerability in ovarian cancer, particularly in ASS1-dysregulated tumors [53,54]. Arginine auxotrophy and metabolic rewiring contribute to tumor survival and immune evasion, making this pathway clinically relevant [53]. The identification of arginine biosynthesis processes supports the presence of tumor-associated metabolic signatures within EV cargo. A prominent enrichment signal was observed for multiple translation-related complexes, including the eukaryotic 80S initiation complex (GO:0033291), translation initiation complex (GO:0070992), eukaryotic translation elongation factor 1 complex (GO:0005853), and 90S preribosome assembly (GO:0034463). Reactome pathways related to maturation of translation-associated proteins were also significantly enriched. Dysregulated translational machinery is a hallmark of malignancy, and enhanced ribosome biogenesis and translation initiation are well-established drivers of ovarian tumor progression [55,56]. The convergence of ribosomal and translational complexes indicates that the EV transcriptome captures a proliferative program characteristic of cancer cells.

Additional enriched processes included positive regulation of intrinsic apoptotic signaling (GO:1902167) and immune-related pathways such as prostaglandin secretion involved in immune response (GO:0090323). Altered apoptotic regulation and inflammatory signaling are central to ovarian cancer pathogenesis and tumor microenvironment remodeling [57,58]. These findings suggest that EV mRNAs not only reflect tumor-intrinsic metabolic and translational programs but also immune-modulatory and stress-response pathways. Collectively, pathway enrichment analysis indicates that ovarian cancer-associated EV mRNAs are functionally enriched for iron homeostasis, arginine metabolism, ribosome assembly and translation initiation, apoptotic regulation, and inflammatory signaling. These processes are mechanistically consistent with established hallmarks of ovarian cancer, supporting the biological relevance of the identified EV expression program.

### 4.2. Validation

To further support the robustness of the identified EV mRNA candidates, we performed biological validation using droplet digital PCR (ddPCR) on a subset of selected transcripts, and the corresponding results have been included as Supplementary Materials File S1. In addition, we performed a supervised differential expression analysis using DESeq2, and the complete results are provided in Supplementary Table S1: Results_deseq2.csv. Comparison of the DESeq2 and NMF-derived candidate lists demonstrated substantial overlap, including several highly ranked transcripts identified by both approaches, supporting the reproducibility and biological relevance of the identified ovarian cancer–associated EV mRNA signature.

## 5. Discussion

This study demonstrates that EV mRNA expression profiles from ovarian cancer patients contain latent low-rank structure recoverable by unsupervised NMF, with at least one dominant component showing preferential activation in cancer relative to control samples. Recovering cancer-associated transcriptional programs without invoking sample labels during decomposition supports the hypothesis that tumor-associated EV mRNA cargo is not stochastically composed but reflects organized, biologically coherent gene programs [9,25]. The non-negativity constraint of NMF produces additive, parts-based components that capture multivariate co-expression structure invisible to gene-wise supervised tests, making it well-suited to the high feature-to-sample regime of EV transcriptomics [23,45]. Rank selection via joint reconstruction error and cophenetic correlation analysis, combined with bootstrap resampling stability scoring, provided a reproducible and overfitting-resistant framework for component identification and gene prioritization [46,51].

Pathway enrichment of cancer-associated module genes revealed four functional clusters consistent with the transcriptional phenotype of high-grade serous ovarian carcinoma. Enrichment of the ferritin complex and iron ion sequestering activity reflects tumor cell adaptation to oxidative stress through labile iron buffering [59,60], while co-enrichment of arginine biosynthesis and argininosuccinate metabolic processes implicates nitrogen metabolism rewiring and amino acid auxotrophy—recognized features of ovarian tumor metabolism [61]. A coordinated cluster of translational machinery terms, including the eukaryotic 80S initiation complex, 90S preribosome assembly, and translation elongation factor complex, is consistent with mTORC1-driven translational upregulation in aggressive ovarian carcinoma [62,63]. Enrichment of the AP-1 transcription factor complex alongside positive regulation of intrinsic apoptotic signaling reflects the concurrent pro-survival and stress-response transcriptional state associated with chemotherapy adaptation [64,65]. Immune-related terms including prostaglandin secretion and cytochalasin B response implicate tumor inflammatory signaling and cytoskeletal remodeling, the latter being mechanistically relevant to EV biogenesis and vesicle shedding rates [66].

Several limitations should be acknowledged. First, the primary limitation of this study is the small cohort size seven ovarian cancer and two control samples which constrains statistical power, restricts the permutation null distribution, and limits generalizability of the recovered components. Second, reliance on mean expression values may obscure intra-sample variability. Third, EV RNA content may partially reflect passive release of abundant cytoplasmic transcripts (e.g., ribosomal RNAs), complicating interpretation of functional enrichment. Future studies incorporating larger patient cohorts, RNA-seq depth normalization, integration with proteomic EV cargo, and longitudinal sampling will be essential to establish clinical utility.

## 6. Conclusions

This study demonstrates that unsupervised matrix factorization of extracellular vesicle (EV) mRNA profiles can uncover coherent and interpretable expression programs associated with ovarian cancer. By integrating systematic filtering, feature selection, and non-negative matrix factorization (NMF), we identified a cancer-associated EV module enriched for transcripts involved in translation, ribosome biogenesis, iron homeostasis, and metabolic regulation—processes central to tumor growth and cellular adaptation. The predominance of overexpressed transcripts in ovarian samples indicates that EV cargo reflects an activated biosynthetic and metabolic state characteristic of cancer cells. Importantly, the proposed composite ranking framework enabled prioritization of a compact set of candidate transcripts by integrating module structure, gene contribution, expression differences, and model stability. This approach moves beyond conventional differential expression analyses by capturing coordinated transcriptional programs rather than isolated gene-level signals. From a methodological perspective, this work highlights the utility of unsupervised learning for extracting structured, biologically meaningful patterns from small, high-dimensional datasets without reliance on class labels, thereby reducing the risk of overfitting. Although validation in larger and independent cohorts is required, the proposed framework provides a robust and generalizable strategy for feature extraction and candidate prioritization. Overall, these findings support the application of unsupervised matrix factorization for EV-based biomarker discovery and broader transcriptomic data analysis.

## Figures and Tables

**Table 1:** Model selection metrics for non-negative matrix factorization across candidate values of K. Reconstruction error decreased monotonically with increasing K, while component stability remained maximal across runs. K = 6 was selected based on optimal stability and lowest reconstruction error, supporting robust and parsimonious decomposition of the EV mRNA matrix.

K	Reconstruction Error	Stability Mean	Stability SD
2	11.74	1.0	1.29 × 10^−16^
3	9.07	1.0	5.14 × 10^−17^
4	7.05	1.0	1.07 × 10^−16^
5	5.73	1.0	1.45 × 10^−16^
6	4.46	1.0	1.54 × 10^−16^

**Table 2:** Effect size estimates for NMF-derived modules comparing ovarian cancer and control samples. Module 5 exhibited the largest magnitude separation (Cohen-like effect size = −1.366) and was designated as the cancer-associated module for downstream analysis. The negative direction indicates reduced module activity in ovarian samples relative to controls.

Module	Effect Size (Cohen-like)
Module 1	+0.45
Module 2	+0.44
Module 3	−0.20
Module 4	+0.44
Module 5	−1.37
Module 6	+0.29

## Data Availability

Data will be available on request.

## Supplementary Materials

The following supporting information can be downloaded at: https://www.mdpi.com/article/10.3390/bioengineering13060597/s1, File S1: Biological validation using digital droplet PCR (ddPCR) [67,68,69,70]; Table S1: Results_deseq2; Figure S1: heatmap_by_samples.

## References

[1] Sung H., Ferlay J., Siegel R.L., Laversanne M., Soerjomataram I., Jemal A., Bray F. (2021). Global cancer statistics 2020: GLOBOCAN estimates of incidence and mortality worldwide for 36 cancers in 185 countries. CA A Cancer J. Clin..

[2] Siegel R.L., Miller K.D., Fuchs H.E., Jemal A. (2022). Cancer statistics, 2022. CA A Cancer J. Clin..

[3] Armstrong D.K., Alvarez R.D., Bakkum-Gamez J.N., Barroilhet L., Behbakht K., Berchuck A., Chen L.-M., Cristea M., DeRosa M., Eisenhauer E.L. (2021). Ovarian cancer, version 2.2020, NCCN clinical practice guidelines in oncology. J. Natl. Compr. Cancer Netw..

[4] Cancer Genome Atlas Research Network (2011). Integrated genomic analyses of ovarian carcinoma. Nature.

[5] Konecny G.E., Wang C., Hamidi H., Winterhoff B., Kalli K.R., Dering J., Ginther C., Chen H.-W., Dowdy S., Cliby W. (2014). Prognostic and therapeutic relevance of molecular subtypes in high-grade serous ovarian cancer. J. Natl. Cancer Inst..

[6] Bowtell D.D., Böhm S., Ahmed A.A., Aspuria P.-J., Bast R.C., Beral V., Berek J.S., Birrer M.J., Blagden S., Bookman M.A. (2015). Rethinking ovarian cancer II: Reducing mortality from high-grade serous ovarian cancer. Nat. Rev. Cancer.

[7] Raposo G., Stoorvogel W. (2013). Extracellular vesicles: Exosomes, microvesicles, and friends. J. Cell Biol..

[8] Zhang L., Yu D. (2019). Exosomes in cancer development, metastasis, and immunity. Biochim. Biophys. Acta (BBA)-Rev. Cancer.

[9] Valadi H., Ekström K., Bossios A., Sjöstrand M., Lee J.J., Lötvall J.O. (2007). Exosome-mediated transfer of mRNAs and microRNAs is a novel mechanism of genetic exchange between cells. Nat. Cell Biol..

[10] Wei Z., Batagov A.O., Schinelli S., Wang J., Wang Y., El Fatimy R., Rabinovsky R., Balaj L., Chen C.C., Hochberg F. (2017). Coding and noncoding landscape of extracellular RNA released by human glioma stem cells. Nat. Commun..

[11] Shurtleff M.J., Yao J., Qin Y., Nottingham R.M., Temoche-Diaz M., Schekman R., Lambowitz A.M. (2017). Broad role for YBX1 in defining the small noncoding RNA composition of exosomes. Proc. Natl. Acad. Sci. USA.

[12] Statello L., Maugeri M., Garre E., Nawaz M., Wahlgren J., Papadimitriou A., Lundqvist C., Lindfors L., Collén A., Sunnerhagen P. (2018). Identification of RNA-binding proteins in exosomes capable of interacting with different types of RNA: RBP-facilitated transport of RNAs into exosomes. PLoS ONE.

[13] Vaksman O., Tropé C., Davidson B., Reich R. (2014). Exosome-derived miRNAs and ovarian carcinoma progression. Carcinogenesis.

[14] Runz S., Keller S., Rupp C., Stoeck A., Issa Y., Koensgen D., Mustea A., Sehouli J., Kristiansen G., A1ltevogt P. (2007). Malignant ascites-derived exosomes of ovarian carcinoma patients contain CD24 and EpCAM. Gynecol. Oncol..

[15] Nakamura K., Sawada K., Yoshimura A., Kinose Y., Nakatsuka E., Kimura T. (2016). Clinical relevance of circulating cell-free microRNAs in ovarian cancer. Mol. Cancer.

[16] Subramanian A., Tamayo P., Mootha V.K., Mukherjee S., Ebert B.L., Gillette M.A., Paulovich A., Pomeroy S.L., Golub T.R., Lander E.S. (2005). Gene set enrichment analysis: A knowledge-based approach for interpreting genome-wide expression profiles. Proc. Natl. Acad. Sci. USA.

[17] Langfelder P., Horvath S. (2008). WGCNA: An R package for weighted gene co-expression network analysis. BMC Bioinform..

[18] Love M.I., Huber W., Anders S. (2014). Moderated estimation of fold change and dispersion for RNA-seq data with DESeq2. Genome Biol..

[19] Robinson M.D., McCarthy D.J., Smyth G.K. (2010). edgeR: A Bioconductor package for differential expression analysis of digital gene expression data. Bioinformatics.

[20] Soneson C., Robinson M.D. (2018). Bias, robustness and scalability in single-cell differential expression analysis. Nat. Methods.

[21] Lee D.D., Seung H.S. (1999). Learning the parts of objects by non-negative matrix factorization. Nature.

[22] Cichocki A., Zdunek R., Phan A.H., Amari S. (2009). Nonnegative Matrix and Tensor Factorizations: Applications to Exploratory Multi-Way Data Analysis and Blind Source Separation.

[23] Hamamoto R., Takasawa K., Machino H., Kobayashi K., Sekino Y., Kato T., Ochiya T. (2022). Application of non-negative matrix factorization in oncology: One approach for establishing precision medicine. Brief. Bioinform..

[24] Alexandrov L.B., Nik-Zainal S., Wedge D.C., Aparicio S.A.J.R., Behjati S., Biankin A.V., Bignell G.R., Bolli N., Borg A., Børresen-Dale A.-L. (2013). Signatures of mutational processes in human cancer. Nature.

[25] Gavish A., Tyler M., Greenwald A.C., Hoefflin R., Simkin D., Tschernichovsky R., Darnell N.G., Somech E., Barbolin C., Antman T. (2023). Hallmarks of transcriptional intratumour heterogeneity across a thousand tumours. Nature.

[26] Miceli R.T., Chen T.Y., Nose Y., Greenwald A.C., Wang M.D. (2024). Extracellular vesicles, RNA sequencing, and bioinformatic analyses: Challenges, solutions, and recommendations. J. Extracell. Vesicles.

[27] Jo A., Green A., Medina J.E., Nose Y., Phan J.H., Wang M.D. (2023). Inaugurating high-throughput profiling of extracellular vesicles for earlier ovarian cancer detection. Adv. Sci..

[28] Dogra N., Chen T.Y., Nose Y., Phan J.H., Wang M.D. (2024). Extracellular vesicles carry transcriptional dark matter revealing tissue-specific information. J. Extracell. Vesicles.

[29] Théry C., Witwer K.W., Aikawa E., Alcaraz M.J., Anderson J.D., Andriantsitohaina R., Antoniou A., Arab T., Archer F., Atkin-Smith G.K. (2018). Minimal information for studies of extracellular vesicles 2018 (MISEV2018). J. Extracell. Vesicles.

[30] Théry C., Amigorena S., Raposo G., Clayton A. (2006). Isolation and characterization of exosomes from cell culture supernatants and biological fluids. Curr. Protoc. Cell Biol..

[31] Andrews S. (2010). FastQC: A quality Control Tool for High Throughput Sequence Data (Version 0.11).

[32] Bushnell B. (2014). BBMap: A Fast, Accurate, Splice-Aware Aligner. LBNL Report LBNL-7065E, Lawrence Berkeley National Laboratory. https://escholarship.org/uc/item/1h3515gn.

[33] Dobin A., Davis C.A., Schlesinger F., Drenkow J., Zaleski C., Jha S., Batut P., Chaisson M., Gingeras T.R. (2013). STAR: Ultrafast universal RNA-seq aligner. Bioinformatics.

[34] Frankish A., Diekhans M., Jungreis I., Lagarde J., Loveland J.E., Mudge J.M., Sisu C., Wright J.C., Armstrong J., Barnes I. (2021). GENCODE 2021. Nucleic Acids Res..

[35] Li H., Handsaker B., Wysoker A., Fennell T., Ruan J., Homer N., Marth G., Abecasis G., Durbin R., 1000 Genome Project Data Processing Subgroup (2009). The sequence alignment/map format and SAMtools. Bioinformatics.

[36] Broad Institute (2019). Picard Toolkit (Version 2.20).

[37] Patro R., Duggal G., Love M.I., Irizarry R.A., Kingsford C. (2017). Salmon provides fast and bias-aware quantification of transcript expression. Nat. Methods.

[38] Sha Y., Phan J.H., Wang M.D. (2015). Effect of low-expression gene filtering on detection of differentially expressed genes in RNA-seq data. 2015 37th Annual International Conference of the IEEE Engineering in Medicine and Biology Society (EMBC).

[39] Roberts A.G., Catchpoole D.R., Kennedy P.J. (2018). Variance-based feature selection for classification of cancer subtypes using gene expression data. 2018 International Joint Conference on Neural Networks (IJCNN).

[40] Dhanya R., Paul I.R., Akula S.S., Sivakumar M., Nair J.J. (2020). F-test feature selection in stacking ensemble model for breast cancer prediction. Procedia Comput. Sci..

[41] Sharifai A.G., Zainol Z. (2020). The correlation-based redundancy multiple-filter approach for gene selection. Int. J. Data Min. Bioinform..

[42] Jia Y., Liu H., Hou J., Kwong S. (2020). Semisupervised adaptive symmetric non-negative matrix factorization. IEEE Trans. Cybern..

[43] Gignac G.E., Szodorai E.T. (2016). Effect size guidelines for individual differences researchers. Personal. Individ. Differ..

[44] Reich D., Price A.L., Patterson N. (2008). Principal component analysis of genetic data. Nat. Genet..

[45] Brunet J.P., Tamayo P., Golub T.R., Mesirov J.P. (2004). Metagenes and molecular pattern discovery using matrix factorization. Proc. Natl. Acad. Sci. USA.

[46] Gaujoux R., Seoighe C. (2010). A flexible R package for nonnegative matrix factorization. BMC Bioinform..

[47] Sun N., Zhao X. (2022). Argininosuccinate synthase 1, arginine deprivation therapy and cancer management. Front. Pharmacol..

[48] Torti S.V., Torti F.M. (2013). Iron and cancer: More ore to be mined. Nat. Rev. Cancer.

[49] Pelletier J., Thomas G., Volarević S. (2018). Ribosome biogenesis in cancer: New players and therapeutic avenues. Nat. Rev. Cancer.

[50] Robichaud N., Sonenberg N., Ruggero D., Schneider R.J. (2019). Translational control in cancer. Cold Spring Harb. Perspect. Biol..

[51] Torti F.M., Torti S.V. (2002). Regulation of ferritin genes and protein. Blood.

[52] Stockwell B.R., Angeli J.P.F., Bayir H., Bush A.I., Conrad M., Dixon S.J., Fulda S., Gascón S., Hatzios S.K., Kagan V.E. (2017). Ferroptosis: A regulated cell death nexus linking metabolism and disease. Cell.

[53] Delage B., Fennell D.A., Nicholson L., McNeish I., Lemoine N.R., Crook T., Szlosarek P.W. (2010). Argininosuccinate synthetase expression in the treatment of cancer. Int. J. Cancer.

[54] Ji J.X., Cochrane D.R., Tessier-Cloutier B., Chen S.Y., Ho G., Pathak K.V., Alcazar I.N., Farnell D., Leung S., Cheng A. (2020). Arginine Depletion Therapy with ADI-PEG20 Limits Tumor Growth in Argininosuccinate Synthase-Deficient Ovarian Cancer, Including Small-Cell Carcinoma of the Ovary, Hypercalcemic Type. Clin. Cancer Res..

[55] Ruggero D. (2013). Translational control in cancer etiology. Cold Spring Harb. Perspect. Biol..

[56] Hwang S.P., Denicourt C. (2024). The impact of ribosome biogenesis in cancer: From proliferation to metastasis. NAR Cancer.

[57] Fulda S. (2015). Targeting apoptosis for anticancer therapy. Semin. Cancer Biol..

[58] Kandalaft L.E., Powell D.J., Singh N., Coukos G. (2022). Immunobiology of high-grade serous ovarian cancer: Lessons for clinical translation. Nat. Rev. Cancer.

[59] Barkley D., Moncada R., Pour M., Liberman D.A., Dryg I., Werba G., Wang W., Baron M., Rao A., Xia B. (2022). Cancer cell states recur across tumor types and form specific interactions with the tumor microenvironment. Nat. Genet..

[60] Wang W., Knovich M.A., Coffman L.G., Torti F.M., Torti S.V. (2010). Serum ferritin: Past, present and future. Biochim. Biophys. Acta.

[61] Lieu E.L., Nguyen T., Rhyne S., Kim J. (2020). Amino acids in cancer. Exp. Mol. Med..

[62] Silvera D., Formenti S.C., Schneider R.J. (2010). Translational control in cancer. Nat. Rev. Cancer.

[63] Bhat M., Robichaud N., Hulea L., Sonenberg N., Pelletier J., Topisirovic I. (2015). Targeting the translation machinery in cancer. Nat. Rev. Drug Discov..

[64] Ye N., Ding Y., Wild C., Shen Q., Zhou J. (2014). Small-molecule inhibitors targeting activator protein 1 (AP-1). J. Med. Chem..

[65] Wu H.J., Liu Y.J., Li H.Q., Chen C., Dou Y., Lou H.F., Ho M.S., Li X.M., Gao Z., Duan S. (2014). Analysis of microglial migration by a micropipette assay. Nat. Protoc..

[66] van Niel G., D’Angelo G., Raposo G. (2018). Shedding light on the cell biology of extracellular vesicles. Nat. Rev. Mol. Cell Biol..

[67] Hindson B.J., Ness K.D., Masquelier D.A., Belgrader P., Heredia N.J., Makarewicz A.J., Bright I.J., Lucero M.Y., Hiddessen A.L., Legler T.C. (2011). High-throughput droplet digital PCR system for absolute quantitation of DNA copy number. Anal. Chem..

[68] Byrne J.A., Maleki S., Hardy J.R., Gloss B.S., Murali R., Scurry J.P., Fanayan S., Emmanuel C., Hacker N.F., Sutherland R.L. (2010). MAL2 and tumor protein D52 (TPD52) are frequently overexpressed in ovarian carcinoma, but differentially associated with histological subtype and patient outcome. BMC Cancer.

[69] Mitra A.K., Davis D.A., Tomar S., Roy L., Gurler H., Xie J., Lantvit D.D., Cardenas H., Fang F., Liu Y. (2015). In vivo tumor growth of high-grade serous ovarian cancer cell lines. Gynecol. Oncol..

[70] Barnes B.M., Nelson L., Tighe A., Burghel G.J., Lin I.H., Desai S., McGrail J.C., Morgan R.D., Taylor S.S. (2021). Distinct transcriptional programs stratify ovarian cancer cell lines into the five major histological subtypes. Genome Med..

