# Supplementary material for: Identifying Ovarian Cancer-Associated EV mRNA Expression Profiles Using Unsupervised Machine Learning and Non-Negative Matrix Factorization
Source: Bioengineering (Basel). 2026 May 22;13(6):597. doi: 10.3390/bioengineering13060597 (PMC13295405; doi:10.3390/bioengineering13060597)
Supplement: Supplementary file 1 [file bioengineering-13-00597-s001.zip › bioengineering-4301345-supplementary/bioengineering-4301345-Supplementary File S1 Biological validation using digital droplet.pdf]

bioengineering

MDPI

*Article*

# Identifying Ovarian Cancer-Associated EV mRNA Expression Profiles Using Unsupervised Machine Learning and Non-Negative Matrix Factorization

**Rama Krishna Thelagathoti, Chao Jiang, Dinesh S. Chandel, Wesley A. Tom, Cleo Sarmiento, Appolinaire Olou, Gary Krzyzanowski and M. Rohan Fernando ***

Molecular Diagnostic Research Laboratory, Center for Sensory Neuroscience, Boys Town National Research Hospital, Omaha, NE 68131, USA; ramakrishna.thelagathoti@boystown.org (R.K.T.); chao.jiang@boystown.org (C.J.); dinesh.chandel@boystown.org (D.S.C.); wesley.tom@boystown.org (W.A.T.); cleo.sarmiento@boystown.org (C.S.); gary.krzyzanowski@boystown.org (G.K.); appolinaire.olou@boystown.org (A.O.)

* Correspondence: m.rohan.fernando@boystown.org

## Supplementary File S1: Biological validation using digital droplet PCR (ddPCR)

For biological validation using digital droplet PCR (ddPCR) three genes were selected: ASS1, FTH1, and MAL2 [67]. Total RNA was extracted from seven ovarian cancer and two noncancer cell lines using a column-based GeneJet RNA purification kit (cat. # K0731; Thermo Fisher Scientific, Waltham, MA, USA), following the manufacturer's recommended protocol. Uniform concentrations (100 ng each) of total RNA was reverse transcribed into cDNA using iScript cDNA Synthesis kit (Cat. #1708890; Bio-Rad Laboratories, Inc. Hercules, CA). The ddPCR reactions were prepared with ddPCR Supermix for Probes, gene-specific mRNA TaqMan assays, using 2ul of diluted cDNA in a 20ul ddPCR reaction volume. Samples were partitioned into ~20,000 droplets using a droplet generator and amplified under standard probe-based thermal cycling conditions. Post-amplification, droplets were analyzed by a droplet reader to distinguish positive from negative droplets based on fluorescence. Gene expression levels were calculated using Poisson statistics and run in technical triplicates. Expression values were normalized to a reference gene and reported as absolute copy number or fold-change.

ASS1 levels differed mainly due to three ovarian cancer cell lines—COV362.4, IGROV1, and OVCAR5. These lines showed distinct expression compared with controls, driving the significant separation between cancer and non-cancer groups (shown in Figure 1). FTH1 results were influenced by strong variability between the control samples themselves, making them an inconsistent reference. Among the cancer lines, OVCAR8 showed the largest difference from each control, but when all cancer lines were analyzed together, the overall cancer-versus-control comparison was not significant, indicating that FTH1 expression is highly cell-line–specific. For MAL2, significant cancer-versus-control differences were driven primarily by COV362.4, OVCAR5, and IGROV1. These cell lines showed consistent expression changes relative to controls, resulting in the overall significance observed.

Differential expression of ASS1, FTH1, and MAL2 varies across ovarian cancer cell lines, indicating a cell-line–specific rather than uniform cancer-driven pattern. For ASS1 and MAL2, the differences from controls are primarily driven by COV362.4, IGROV1, and OVCAR5, suggesting links to underlying genotype, mutational background, or tissue-of-origin features [68]. In contrast, FTH1 shows variability even within the control group, making disease-related changes harder to interpret. Although OVCAR8 shows

strong differential expression, significance is lost when cancer lines are pooled, indicating that FTH1 expression is shaped more by individual cell-line biology than by a shared cancer-specific mechanism [69]. Overall, these findings suggest that these genes may act in a context-dependent manner tied to molecular subtype or lineage characteristics [4]. Thus, ASS1, FTH1, and MAL2 may serve as markers of specific ovarian cancer genotypes rather than broad indicators of malignancy. Further work with larger datasets and integrated genomic analyses is needed to clarify these subtype-specific patterns.

## References


67. Hindson, B.J.; Ness, K.D.; Masquelier, D.A.; Belgrader, P.; Heredia, N.J.; Makarewicz, A.J.; et al. High-throughput droplet digital PCR system for absolute quantitation of DNA copy number. *Anal. Chem.* **2011**, *83*, 8604–8610. https://doi.org/10.1021/ac202028g.

68. Byrne, J.A.; Maleki, S.; Hardy, J.R.; Gloss, B.S.; Murali, R.; Scurry, J.P.; et al. MAL2 and tumor protein D52 (TPD52) are frequently overexpressed in ovarian carcinoma, but differentially associated with histological subtype and patient outcome. *BMC Cancer* **2010**, *10*, 497. https://doi.org/10.1186/1471-2407-10-497.

69. Mitra, A.K.; Davis, D.A.; Tomar, S.; Roy, L.; Gurler, H.; Xie, J.; et al. In vivo tumor growth of high-grade serous ovarian cancer cell lines. *Gynecol. Oncol.* **2015**, *138*, 372–377. https://doi.org/10.1016/j.ygyno.2015.05.040.

70. Barnes, B.M.; Nelson, L.; Tighe, A.; Burghel, G.J.; Lin, I.H.; Desai, S.; et al. Distinct transcriptional programs stratify ovarian cancer cell lines into the five major histological subtypes. Genome Med. 2021, 13, 140. https://doi.org/10.1186/s13073-021-00952-5.


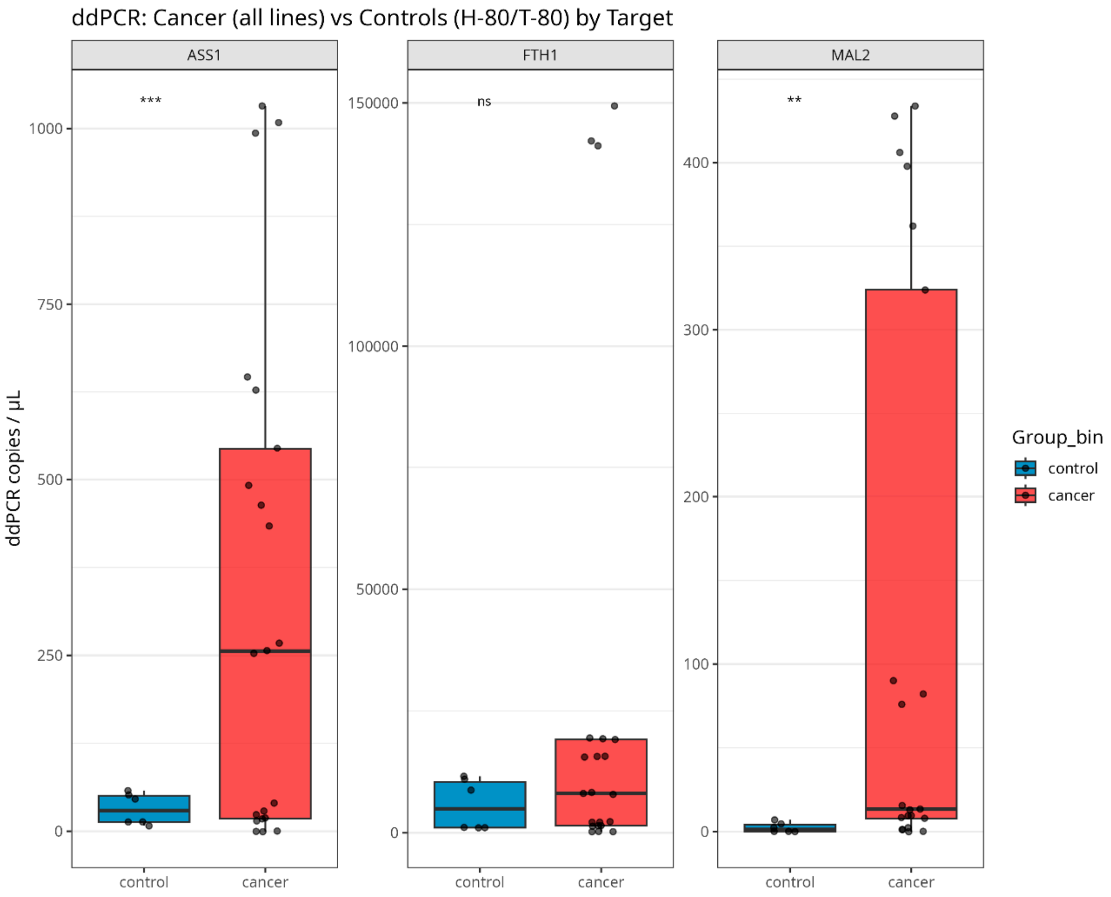


**Figure S2. ddPCR validation of ASS1, FTH1, and MAL2 expression in ovarian cancer cell lines.** Gene ASS1: COV362.4, IGROV1, OVCAR5 are driving differences from controls.; Gene FTH1: controls are significantly different from one another, and only OVCAR8 is really driving the large differential and it is not significant when all cancer lines are grouped.; Gene MAL2: COV362.4, OVCAR5, and IGROV1 are driving the significance between cancer and control. Overall, differential

expression of ASS1, FTH1, and MAL2 varies across ovarian cancer cell lines, indicating a cell-line–specific rather than uniform cancer-driven pattern.

**Disclaimer/Publisher's Note:** The statements, opinions and data contained in all publications are solely those of the individual author(s) and contributor(s) and not of MDPI and/or the editor(s). MDPI and/or the editor(s) disclaim responsibility for any injury to people or property resulting from any ideas, methods, instructions or products referred to in the content.
